# An uncharacterized gene *Lb1G04794* from *Limonium bicolor* promotes salt tolerance and trichome development in *Arabidopsis*

**DOI:** 10.3389/fpls.2022.1079534

**Published:** 2022-12-08

**Authors:** Xiangmei Jiao, Boqing Zhao, Baoshan Wang, Fang Yuan

**Affiliations:** Shandong Provincial Key Laboratory of Plant Stress, College of Life Sciences, Shandong Normal University, Ji’nan, Shandong, China

**Keywords:** *Limonium bicolor*, *Arabidopsis thaliana*, root length, trichome, salt gland, salt tolerance, biomass

## Abstract

Halophytes can grow and reproduce in high-salinity environments, making them an important reservoir of genes conferring salt tolerance. With the expansion of saline soils worldwide, exploring the mechanisms of salt tolerance in halophytes and improving the salt tolerance of crops have become increasingly urgent. *Limonium bicolor* is a halophyte with salt glands that secrete excess Na^+^ through leaves. Here, we identified an uncharacterized gene *Lb1G04794*, which showed increased expression after NaCl treatment and was high during salt gland development in *L. bicolor*. Overexpression of *Lb1G04794* in *L. bicolor* showed promoted salt gland development, indicating that this gene may promote salt gland differentiation. Transgenic Arabidopsis strains overexpressing *Lb1G04794* showed increased trichomes and decreased root hairs under normal conditions. Compared with wild type (WT), root growth in the transgenic lines was less inhibited by NaCl treatment. Transgenic seedlings accumulated less fresh/dry weight reductions under long-term salt treatment, accompanied by lower Na^+^ and malondialdehyde accumulation than WT, indicating that these transgenic lines behave better growth and undergo less cellular damage under NaCl stress. These results were consistent with the low expression levels of salt-tolerance marker genes in the transgenic lines upon salt stress. We conclude that the unknown gene *Lb1G04794* positively regulated salt gland development, and promoted salt tolerance of Arabidopsis, offering a new direction for improving salt tolerance of non-halophytes and crops.

## 1 Introduction

According to incomplete statistics from UNESCO and FAO, there are 953 million hectares of saline land in the world, 10% of which are in China. High ion concentrations and low water potential hinder normal plant growth and development, thus reducing crop yields and limiting food production (Boyer, 1982; Yuan et al., 2013). Saline lands affect the surrounding ecological environment, and intensifies with the expansion of human activities (Munns and Tester, 2008; Song et al., 2020; Zheng et al., 2020). The current decrease in arable land and the increase in the human population underscore the urgent need to effectively utilize saline land to expand usable land for the cultivation of adapted crops (Munns and Tester, 2008; Guo et al., 2017; Ma et al., 2020).

Although a number of physical and chemical methods such as salt washing, concealed pipe and chemical modification are currently used to remediate high-salinity soils, these methods are costly, have poor durability, and are prone to several environmental problems, which are likely to cause secondary salinization (Rasool et al., 2013). A more effective and sustainable transformation method should therefore call upon biological means of improving saline soils. Many salt tolerance genes have been isolated and introduced into various plant species to improve their tolerance to salt stress, which can be applied to valorize saline soils. Halophytes that can grow naturally on saline land have great potential in the biological improvement of saline-alkali land (Yuan et al., 2019b), which can be fueled by research on halophytes and their underlying salt tolerance mechanisms and associated key genes. The physiological response and adaptation mechanism of halophytes to soil salinity (Lu et al., 2022) have been extensively studied and confirmed.

Halophytes can improve the ecological environment and utilization degree of saline land by preserving soil and water, increasing vegetation coverage, and increasing the abundance of surface animals and microorganisms. Halophytes can complete their life cycle under conditions as extreme as 200 mM NaCl, using one of three strategies: secreting salt back into the environment (recretohalophytes), salt compartmentalization into cell vesicles (euhalophytes), or preventing salt from entering cells (pseudohalophytes) (Yuan et al., 2013; Song and Wang, 2015; Guo et al., 2020a). Among these three types of halophytes, recretohalophytes have a specific salt tolerance mechanism and can typically secrete Na^+^ out of plant cells to avoid salt damage.

Several genes participating in salt tolerance and encoding proteins with distinct functional domains have been characterized in halophytes. *SUPER SENSITIVE TO ABA AND DROUGHT2* (*LbSAD2*) significantly increased salt resistance of Arabidopsis (*Arabidopsis thaliana*) seedlings by reducing root hair development and sensitivity to the abscisic acid (ABA), the physiological indexes of overexpressed lines were significantly better than those of WT under salt treatment (Xu et al., 2021). Similarly, *Limonium bicolor HELIX-LOOP-HELIX* (*LbHLH*) increased salt tolerance by reducing root hair development and increasing osmotic resistance when overexpressed in Arabidopsis (Wang et al., 2021). *TRIPTYCHON* (*LbTRY*) increased salt sensitivity when overexpressed in Arabidopsis by increasing root hair number and changing osmotic homeostasis (Leng et al., 2021). Analysis of salt tolerance mechanisms in the recretohalophyte *Limonium bicolor* established that the loss and modification of key genes initiating trichomes in other species have led to the development of salt glands instead of trichomes in this plant species (Yuan et al., 2022). Additional salt tolerance genes have been described in other species as well: The heterologous expression of *NAM, ATAF1/2, CUC2 17* (*LpNAC17*) from coral lily (*Lilium pumilum*) in *Nicotiana benthamiana* improved salt tolerance by shortening root length and line size (Cui, 2021), and *ALTERNATIVE OXIDASE2* from Chinese dwarf cherry (*Cerasus humilis*) (*ChAOX2*) transformation of *Arabidopsis thaliana* enhanced plant respiration, decreased ion leakage, increased proline content, and decreased the accumulation of reactive oxygen species to improve salt tolerance (Zhao, 2020). Protein–protein interaction can also significantly affect salt tolerance. For example, the transcription factors MdWRKY55 and MdNAC17-L from apple (*Malus domestica*) enhanced salt tolerance by activating the transcription of the *Na^+^/H^+^ exchanger 1* gene *MdNHX1* (Su et al., 2022). A network of salt tolerance genes is gradually emerging from the integration and exploration of the above genes and their mechanisms.

Analysis of the mechanisms involved in salt tolerance includes the characterization of proteins with unknown or poorly characterized functional domains. For example, the gene *Lb1G04202* conferred a stronger salt tolerance at the seedling stage when overexpressed in Arabidopsis and participated in salt tolerance by promoting proline biosynthesis (Wang et al., 2022). Mutants of *At5G45480* were more sensitive to osmotic stress; an analysis of differentially expressed genes between the mutant and WT revealed that the expression levels of genes related to substance biosynthesis and transport and related to protein translation and activity were closely related to *At5G45480* function (Zhu, 2014). The overexpression of *Lb2G14763* in *Arabidopsis thalian*a resulted in the greater accumulation of Na^+^ and lower expression of salt-resistant genes compared to nontransgenic controls, resulting in the negative regulation of salt tolerance. Transgenic *Arabidopsis* lines overexpressing *Lb7G32827* and *Lb3G18904* showed more salt tolerance at the seedling stage than WT, and the expression of salt-tolerant marker genes was significantly increased, contributing to higher salt tolerance (Jing, 2021). A gene was isolated from an expressed sequenced tag (EST) library generated from mustard (*Brassica rapa*) exposed to 200 mM NaCl; The superepitope strain of this gene grew better than the WT under salt treatment (Wei, 2006). As more genes with unknown function are discovered and characterized, the plant salt tolerance pathway becomes more complex.

Salt glands are a typical salt secretory epidermal structure that are present in 68 species, including sea lavender (*L. bicolor*) (Li et al., 2020; Lu et al., 2020). *L. bicolor* belongs to the Plumbaginaceae (Yuan et al., 2016a; Leng et al., 2018; Yuan et al., 2018; Leng et al., 2019a; Leng et al., 2019b; Guo et al., 2020b; Gao et al., 2021) and is a unique recretohalophyte with a sequenced genome that constitutes an essential genetic resource for improving salt tolerance in crops. Salt gland differentiation is the earliest visible sign on the epidermis, even before the development of stomata and other epidermal structures. Yuan et al. (2015) divided the differentiation and development time of epidermal cells into five stages, namely, undifferentiated stage (Stage A), salt gland differentiation stage (Stage B), stomatal differentiation stage (Stage C), pavement cell differentiation stage (Stage D) and maturation stage (Stage E) through observation on the first true leaf of *L. bicolor*. A previous transcriptome analysis of salt gland development and salt secretion (Yuan et al., 2015; Yuan et al., 2016b) identified the gene *Lb1G04794*, which encodes a protein with an uncharacterized domain, with high expression during salt gland development. As a typical halophyte with high salt tolerance and special salt secretion structure, *L. bicolor* has not been thoroughly studied. With the in-depth study of its transcriptome (Yuan et al., 2022), the mechanism of salt resistance will be further clarified. As an unknown gene, *Lb1G04794* is a further exploration of the mechanism of salt gland development and an effective means to perfect the mechanism of salt tolerance. To explore the function of this novel gene, we overexpressed *Lb1G04794* in Arabidopsis, which revealed its positive role in salt responses, suggesting that upregulated genes in response to *Lb1G04794* overexpression may be related to salt tolerance. *Lb1G04794* overexpression may therefore offer a means to improving salt tolerance in crops.

## 2 Materials and methods

### 2.1 Plant materials and growth conditions

Seeds of *L. bicolor* were collected from the saline inland environment (N37°20’; E118°36’) in the Yellow River Delta, Shandong, China. The seeds were well dried and stored at 4°C until use. The seeds were washed with sterile deionized water after surface disinfection with 70% (v/v) ethanol for 5 min and 6% (v/v) sodium hypochlorite (Sigma, United States) for 15–20 min. Surface-sterilized seeds were then sown onto Murashige and Skoog (Murashige and Skoog, 1962) medium (MS medium; adjusted to pH 5.8 with KOH before autoclaving). The plates were incubated at 28 ± 3°C/23 ± 3°C (day/night cycle) under a light intensity of 600 µmol/m^2^/s (15-h-light/9-h-dark photoperiod) and 70% relative humidity. Samples were collected and frozen in liquid nitrogen at the undifferentiated stage (5,000 leaves in stage A), the salt gland development stage (4,000 leaves in stage B), and the first true leaf was collected (Yuan et al., 2015). Total RNA was extracted for gene cloning.

The *Arabidopsis thaliana* accession Columbia-0 (Col-0) was used for heterologous overexpression of *Lb1G04794*. Seeds were first surface sterilized three times with 75% (v/v) ethanol for 4 min, during which a full eddy was applied, followed by 95% (v/v) ethanol for 1 min, repeated three times, with a full rinse with sterile water four times. The seeds were then sown onto half-strength MS medium (pH 5.8). After stratification at 4°C for 2 days, the plates were released at 22°C/18°C (day/night) under a light intensity of 150 mol/m^2^/s, relative humidity of 70%, and a light cycle of 16 h light/8 h dark (Sui et al., 2017). Seedlings were transferred into small pots (10 cm in diameter and 8 cm in height) containing mixed soil (soil:vermiculite:perlite, 3:1:1) after 1 week and were allowed to grow under the same growth conditions for transformation and treatment.

### 2.2 Cloning of full-length cDNA and bioinformatic analysis of Lb1G04794

The first true leaves over the A-E period of *L. bicolor* leaf development were collected, frozen in liquid nitrogen, and stored at –80°C before total RNA extraction according to Yuan. (Yuan et al., 2015). A ReverTra Ace^®^ qPCR RT Kit (Japan TOYOBO CO, LTD) was used for reverse transcription to obtain cDNA for each developmental stage. Based on Iso-seq transcriptome data from *L. bicolor*, the primers *Lb1G04794*-S and *Lb1G04794*-A for *Lb1G04794* were designed using Primer Premier 5.0, and the full-length coding sequence was amplified by PCR (**Supplementary Table 1**).

DNA and protein sequences were compared using DNAman and DNAstar. After BLAST with *Lb1G04794* as a query at the National Center for Biotechnology Information (NCBI), 33 related proteins were selected for phylogenetic tree construction, using MEGA and the ClustalX adjacency method. The percentage support at each node was determined from at least 1,000 bootstrap replicates. Using the ProtParam tool in ExPASy online software, the physicochemical properties of each protein were predicted. The hydrophilicity and hydrophobicity of all proteins were analyzed by prot-Scala in ExPASy. The software tools Signal4.14, ExPASy and Swiss-Model were used to predict the secondary and tertiary structures of the proteins and the presence of signal peptides.

### 2.3 Subcellular localization of Lb1G04794

A 2×Taq Plus Master Mix II and the primer pair *Lb1G04794* 1300-S and *Lb1G04794* 1300-A (**Supplementary Table 1**) were used to amplify the full-length coding sequences with homologous terminal vectors. The *Lb1G04794* coding sequence was cloned into the pCAMBIA 1300 vector containing the cauliflower mosaic virus (CaMV) 35S promoter, the hygromycin resistance gene, and the green fluorescent protein sequence (*GFP*). The resulting pCAMBIA 1300-*Lb1G04794* vector was transformed into onion (*Allium cepa*) epidermal cells using Agrobacterium (*Agrobacterium tumefaciens*) strain GV3101 (Sun et al., 2007). The fluorescence signal of the GFP fusion protein was detected with a confocal microscope (TCS S8 MP two-photon confocal laser scanning microscope, Leica, Germany). Staining with 4′,6-diamidino-2-phenylindole (DAPI) was used to show the nucleus under 358-nm excitation.

### 2.4 Transcriptional activation assay of Lb1G04794 in yeast cells

ClonExpress^®^ II was used to clone the coding sequence of *Lb1G04794* into the vector pGBKT7/BD *via* the NdeI restriction site (**Supplementary Table 1**). The three vector pairs pGADT7-T+pGBKT7-*Lb1G04794* (experimental group), pGADT7-T+pGBKT7-lam (negative control), and pGADT7-T+pGBKT7-53 (positive control) were introduced into Y2H Gold yeast cells through the yeast Maker transformation system. Yeast colonies were selected on synthetic defined (SD) medium lacking Trp (SD –Trp) for 3 days. Transcriptional activity was evaluated according to yeast growth on SD –Trp –Leu medium at 30°C for 2 days (Guo et al., 2013). β-Galactosidase activity was determined by growth on SD –Trp –Leu –Ade –His medium containing X-α-gal (Han et al., 2019).

### 2.5 Expression analysis in different tissues in *Limonium bicolor*

According to transcriptome deep sequencing (RNA-seq) results of *L. bicolo*r samples at different developmental stages, *Lb1G04794* was expressed at different levels across different developmental stages (Yuan et al., 2015). To validate these results, samples were collected from the first true leaf at the A and B stages (undifferentiated stage, 4–5 days after sowing; salt gland differentiation stage, 6–7 days after sowing, using 4,000 leaves), the C and D stages (stomatal differentiation, 8–10 days; epidermal cell differentiation stage, 11–13 days, 2,000 leaves), the E stage (mature young stage, 14 days, 500 leaves), old leaves (20 days) and the E stage petioles and roots. Meanwhile, we also collected true leaf materials treated with 300 mM NaCl for 14 days. Total RNA was extracted from the above materials. A separate set of seedlings was treated with 25 mg/L salicylic acid, 0.1 mg/L methyl jasmonate, or 300 mM NaCl, then sampled at 0, 6, 12, 24, 48, and 72 h. Beacon Designer Free Edition software (Version 7.8) was used to design the primers for quantitative PCR (qPCR) of *Lb1G04794*, using *LbTUBULIN* as internal control (**Supplementary Table 1**). PCR thermal cycling conditions were as follows: denaturation at 95°C for 5 min, followed by 40 cycles of denaturation at 94°C for 20 s, annealing at 58°C for 15 s, extension at 65°C for 15 s. Three biological replicates were analyzed. Relative expression levels were calculated according to the formula 2^−ΔΔC(T).^

### 2.6 Analysis of the tissue-specific expression of Lb1G04794 in Arabidopsis

The first true leaves of *L. bicolor* seedlings were collected, and genomic DNA was extracted by the cetyltrimethylammonium bromide (CTAB) method. The *Lb1G04794* promoter sequence was identified in the *L. bicolor* genome sequence (Lescot et al., 2002), and specific primers (*Lb1G04794*-P-S and *Lb1G04794*-P-A) were designed using Primer Premier 5.0 (**Supplementary Table 1**). The promoter fragment was amplified by PCR with a 2×Taq Plus Master Mix and cloned, as detailed below.

The CaMV 35S promoter in pCAMBIA3301-35S-GUS vector was excised by digestion with HindШ/NcoI to linearize the vector. The *Lb1G04794pro:GUS* reporter construct was obtained by linking the linearized vector and the *Lb1G04794* promoter with ClonExpress^®^ II. The resulting construct was transformed into wild-type Arabidopsis plants by Agrobacterium-mediated transformation (strain GV3101). Transgenic plants were obtained by selection on basta. A GUS staining kit (Zhongkelitai Biological Technology Co, LTD; Cat No : RTU4032)was used for staining: a 50× X-Gluc concentrated solution was diluted 50 times with GUS staining buffer. The prepared materials were soaked in GUS staining solution overnight at 25–37°C. All materials were then transferred into anhydrous ethanol for chlorophyll clearing two to three times until the negative control material turned white. GUS staining was observed with the naked eye or under a microscope.

### 2.7 Vector construction and transformation of arabidopsis Col-0

After linearization of pCAMBIA3301-35S-GUS vector by digestion with NcoI to remove the 35S promoter coding sequence, the *Lb1G04794* coding sequence was amplified by PCR with the primers *Lb1G04794* 3301-S and *Lb1G04794* 3301-A (**Supplementary Table 1**) and cloned into the linearized vector with the ClonExpress^®^ II recombination reaction system to generate *35S*: *Lb1G04794*. The resulting construct was introduced into Agrobacterium strain GV3101 and transformed into Arabidopsis Col-0 by Agrobacterium-mediated floral dipping (Clough and Bent, 1998). After three generations of selection for herbicide resistance, homozygous *35S*:*Lb1G04794* lines were identified. Genomic DNA was extracted for PCR with the primers pCAMBIA-S and *Lb1G04794*-A to confirm transgenic plants harboring the overexpression construct (**Supplementary Table 1**). Total RNA of *35S*:*Lb1G04794* transgenic lines was extracted with a FastPure Plant Total RNA Isolation Kit (Vazyme, China). The expression levels of *Lb1G04794* were measured in the transgenic lines by RT-qPCR using *Lb1G04794* RT-S and *Lb1G04794* RT-A primers (**Supplementary Table 1**), using Arabidopsis *ACTIN2* as internal control (primers ACTIN2 sense and ACTIN2 anti). The expression of each transgenic line was repeated three times. The line with the lowest *Lb1G04794* expression level (line OE14) was used as the control (with relative expression level set to 1) to calculate the relative expression level of *Lb1G04794* in the other overexpression lines (Leng et al., 2021).

### 2.8 Overexpression transformation of Lb1G04794 in *Limonium bicolor*

pCAMBIA3301-35S-GUS and pCAMBIA3301-35S-*Lb1G04794*-GUS was transferred into *Agrobacterium* EHA105 and used to infect *L. bicolor* referring to Yuan et al. (2104). After the shoot regeneration, the regenerated leaves were transferred to the root regeneration medium. After screened in hygromycin for two weeks, the regenerated seedlings were used for qRT-PCR to verify the expression level in overexpression line. Then, the leaves were fixed in Carnoy (ethanol: acetic acid, 3:1) for 12 h and decolorized with 70% ethanol for 12 h, and finally decolorized with Hoyers’ solution (chloral hydrate saturated with lactic acid solution). The structure and morphology of the salt glands were observed under the excitation light of 330–380 nm under the fluorescence microscope, and then the number of salt glands in a single leaf was counted.

### 2.9 Observation of root hairs and trichomes in transgenic lines under salt treatment

The phenotypes of one-week-old T_3_ homozygous seedlings were observed using an anatomical microscope (Nikon, Japan). After the first pair of true leaves had fully expanded, the total number of trichomes was counted on the first pair of rosette leaves, with 20 seedlings analyzed for each line. The number and length of root hairs from five OE-*Lb1G04794* seedlings were scored, with 20 seedlings per line.

### 2.10 Effect of NaCl concentration on salt tolerance of different transgenic lines: Root length and physiological indicators

Three Arabidopsis transgenic lines overexpressing *Lb1G04794* at high, medium, or low expression levels, together with Arabidopsis wild-type Col-0, were treated with NaCl. All seeds were sown on half-strength MS medium alone or containing different concentrations of NaCl (50, 100, and 150 mM). After stratification for 2–3 days, the plates were released into tissue culture chambers. After 24 h, the germination percentage was scored as the emergence of the radicle through the seed coat. Thegermination percentage was calculated as follows: germination percentage (%) = number of germinated seeds/total seeds × 100%. Since the germination percentage of overexpressing lines was significantly lower than that of Col-0, the relative germination percentage of impermeable salt treatment was calculated as follows: (germination percentage under control − germination percentage under NaCl treatment)/germination percentage under control × 100% on half-strength MS medium.

All seeds were evenly sown on medium with different NaCl concentrations (0, 50, 100, and 150 mM) as three replicates. Seedlings were photographed after 5 days with an anatomical microscope, and root length was measured in ImageJ. As the relative inhibition rate of root length elongation was significantly lower than that of the WT, the relative shortening rate was also calculated. The relative inhibition rate of root length elongation was calculated as root length under control – root length under NaCl treatment)/root length under control × 100% on half-strength MS medium.

According to Han et al. (Han et al., 2019) and Guo et al. (Guo, 2017), 7-day-old Arabidopsis seedlings were transferred to nutrient soil and then treated with different concentrations of salt for 1 week. The fresh weight and various physiological indicators were determined after salt treatment for 1 week. The relative decrease in fresh weight was calculated as (fresh weight under control – fresh weight under NaCl treatment)/fresh weight under control × 100%. The relative reduction in dry weight was calculated as (dry weight under control – the dry weight under NaCl treatment)/dry weight under control × 100%. To determine the contents of various small molecules, 0.5 g of seedlings growing under different NaCl concentrations was collected and Na^+^, K^+^, malondialdehyde (MDA), and proline contents were measured. Ion concentrations were determined using a flame photometer (M410, Sherwood, United Kingdom). Five replicates were performed for each line.

### 2.11 Determination of plant hormone contents in Lb1G04794 transgenic Arabidopsis

Endogenous phytohormones were extracted from 7-day-old Arabidopsis seedlings by the isopropanol-water-hydrochloric acid method. Endogenous phytohormone contents were determined with an Agilent 1290 high-performance liquid chromatography (HPLC) tandem AB Sciex QTRAP 6500^+^ mass spectrometer, and internal standard substances were added during extraction. Since the growth and development of overexpression lines were clearly weaker than those of the WT, the contents of indole-3-acetic acid (IAA) and ABA were specifically targeted for determination.

### 2.12 Analysis of salt resistance marker gene expression in Lb1G04794 transgenic Arabidopsis

Total RNA was extracted from Arabidopsis seedlings grown on half-strength MS medium for 5 days and transplanted to soil for 2 weeks. The expression of six stress-related genes, *SALT OVERLY SENSITIVE1* (At2G01980, *AtSOS1*), *SOS2* (At1G01140), *SOS3* (At5G35410), *HIGH-AFFINITY K^+^ TRANSPORTER1* (At4G1030, *AtHKT1*), *Na^+^/H^+^ EXCHANGER1* (At5G27150, *AtNHX1*), and *GST CLASS TAU5* (At2G29450, *AtGSTU5*), was determined by RT-qPCR (**Supplementary Table 1**). Three biological replicates were performed. The formula 2^−ΔΔC(T)^ was used to calculate relative expression, with *ACTIN2* used as internal reference.

### 2.13 Statistical analysis

Statistical analysis was performed using SPSS at *P* = 0.05 (Duncan’s multiple range tests). Analysis of variance (ANOVA) with orthogonal contrasts and mean comparison procedures was used to detect differences between the treatments.

## 3 Results

### 3.1 Characteristics and expression pattern of Lb1G04794

**Figure 1:**
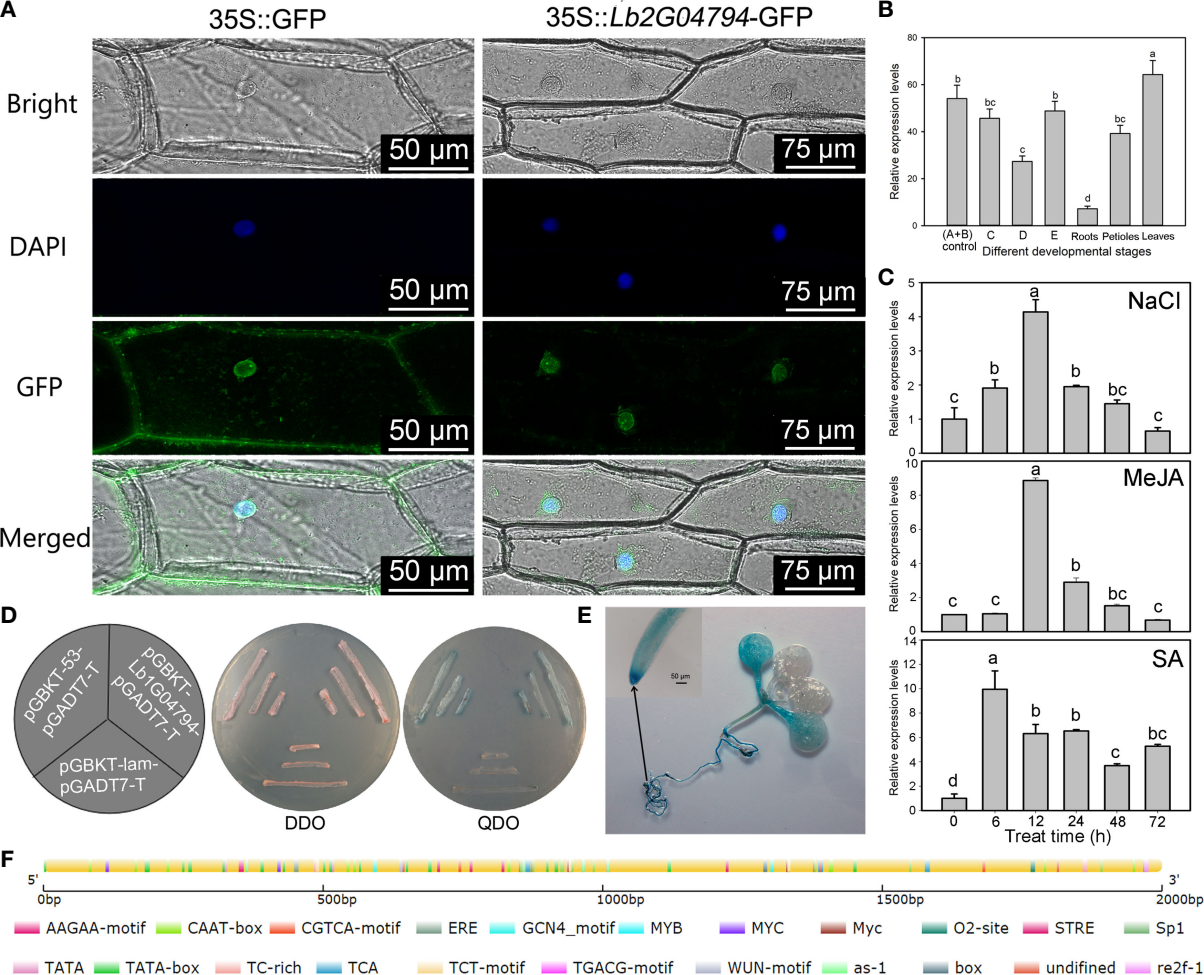
Expression pattern of *Lb1G04794* and subcellular localization of its encoded protein. **(A)** Subcellular localization of the protein encoded by the unknown gene *Lb1G04794* in onion epidermal cells transiently transformed with *35S*:*Lb1G04794*-*GFP via* Agrobacterium. pCAMBIA 1300-35S-sGFP (*35S:GFP*) was used as an empty control vector. Scale bar, 100 µm in *35S:GFP*, 250 µm in *35S*:*Lb1G04794*-*GFP*. **(B)** Expression of *Lb1G04794* in *L. bicolor* at different developmental stages. Stage A, undifferentiated, 4–5 days after sowing; stage B, salt gland differentiation, 6–7 days after sowing; stage C, stomatal differentiation, 8–10 days after sowing; stage D, epidermal differentiation, 11–16 days after sowing; stage E, mature, more than 17 days after sowing. Old leaves, >20 days old; petiole, base of stage-E leaf; root, root of stage-E seedling. Data are means of three replicates ± SD; different lowercase letters indicate significant differences at *P* = 0.05 according to Duncan’s multiple range test. **(C)** Changes in *Lb1G04794* expression levels under different treatments: NaCl, MeJA, and SA for 6, 12, 24, 48, and 72 h. Data are means of three replicates ± SD; different lowercase letters indicate significant differences at *P* = 0.05 according to Duncan’s multiple range test. **(D)** The protein encoded by *Lb1G04794* self-activates when expressed in yeast. Colonies were grown on synthetic defined (SD) medium –Trp –Leu (SD –Trp –Leu) and SD –Trp –Leu –Ade –His+X-α-gal. pGADT7-T+pGBKT7-*Lb1G04794*, experimental group; pGADT7-T+pGBKT7-53, positive control; pGADT7-T+pGBKT7-lam, negative control. **(E)** GUS staining results of the *Lb1G04794* promoter driving GUS expression in Arabidopsis transgenic lines (7 days after germination). **(F)** Schematic diagram of the *Lb1G04794* promoter, as predicted using PlantCARE (Lescot et al., 2002 (http://bioinformatics.psb.ugent.be/webtools/plantcare/html/); the map was drawn using CSDS 2.0 (Hu et al., 2015) (http://gsds.gao-lab.org/). Different colors indicate different *cis*-elements.

Based on the full-length sequence of *Lb1G04794*, we cloned a 1,074-bp open reading frame encoding a 357–amino acid protein with a predicted molecular weight of 39,928.25 Da and an isoelectric point (PI) of 6.36 (**Supplementary Figure 1A**). The gene sequence was verified to be consistent with the genomic data by sequencing. Of these 357 amino acids, 110 were hydrophobic, accounting for 30.8% of the total amino acid number, and 247 were hydrophilic (or 69.2%). The predicted protein had an aliphatic index of 65.01, indicating that the protein encoded by *Lb1G04794* is a hydrophilic protein without a transmembrane helical structure (**Supplementary Figure 1B**). We identified no homologous protein for Lb1G04794 in Arabidopsis. We thus used a BLAST search at NCBI with the predicted Lb1G04794 protein sequence as query (**Supplementary Figure 2**). We determined that the protein encoded by *Lb1G04794* is a hypothetical protein with no transmembrane domain (**Supplementary Figure 1C**), no signal peptide (**Supplementary Figure 1D**), and no conserved domain (**Supplementary Figure 1E**), making its function completely unknown. An analysis of the *Lb1G04794* promoter (2,000 bp upstream of the ATG) identified a core promoter element and a typical *cis*-element involved in light responses, a *cis*-element associated with methyl jasmonate (MeJA) responses (CGTCA-motif), a *cis*-acting element involved in salicylic acid (SA) reaction (TCA-element), and a *cis*-acting element involved in defense and stress response (TC-rich repeats) (**Figure 1F**). We generated transgenic Arabidopsis lines harboring the *Lb1G04794* promoter driving the transcription of the *β-GLUCURONIDASE* (*GUS*) reporter gene; we observed GUS staining in cotyledons and roots, especially in the root tip of these transgenic reporter lines (**Figure 1E** **)**.

### 3.2 Lb1G04794 localizes to the nucleus and has self-activation activity

To determine the subcellular localization of the protein encoded by *Lb1G04794*, we transiently transformed onion epidermal cells with Agrobacterium harboring the vector p1300-*Lb1G04794*, encoding a fusion protein between GFP and the protein encoded by *Lb1G04794*. Observations with a two-photon fluorescence inverted microscope showed that GFP-*Lb1G04794* localizes specifically in the nucleus (**Figure 1A**), while the empty vector overexpressing free *GFP* resulted in green fluorescence in both the nucleus and the cytoplasm. We also studied the expression of *Lb1G04794* in *L. bicolor* at different developmental stages (**Figure 1B**) and under different treatments (**Figure 1C**). *Lb1G04794* was highly expressed during stages A and B, responded to salt treatment, and was most highly expressed in leaves compared to other tissues. We also observed that *Lb1G04794* expression responded to NaCl, SA, and MeJA treatments, with a peak in *Lb1G04794* transcript levels 6–12 h into the treatment, followed by a gradual decrease back to normal levels (**Figure 1C**). Finally, we determined that the protein encoded by *Lb1G04794* displays self-activation activity in yeast when fused to the GAL4 DNA-binding domain (**Figure 1D**).

### 3.3 *Lb1G04794* participated in salt gland development of *Limonium bicolor*

**Figure 2:**
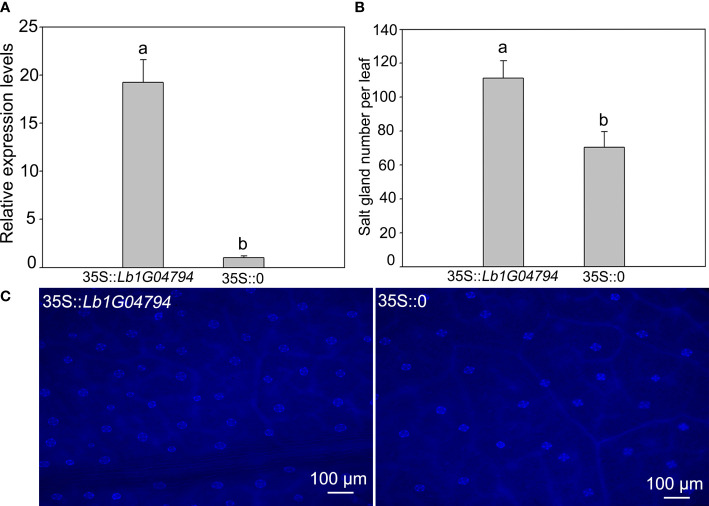
The salt gland phenotypes and quantification after overexpressing *Lb1G04794* in *L. bicolor*. **(A)** The expression level of overexpression line (35S::*Lb1G04794*) and control (35S::0). **(B)** The total number of salt glands per leaf. Data are means ± SD of three seedlings; different letters indicate significant differences at *P* = 0.05 according to Duncan’s multiple range test. **(C)** The salt gland phenotypes in overexpression line and control under 330–380 nm microscope.

The *Lb1G04794* was overexpressed in *L. bicolor* (**Figure 2**), after expression level verification (**Figure 2A**), compared with the control transformed with the empty vector, overexpression of *Lb1G04794* can significantly enhanced salt gland development (**Figures 2B, C**). This indicated that *Lb1G04794* may participate in promoting salt gland differentiation.

### 3.4 Transgenic arabidopsis lines develop more trichomes and fewer root hairs

We isolated Arabidopsis transgenic lines overexpressing *Lb1G04794* from the cauliflower mosaic virus (CaMV) 35S promoter. We confirmed the presence of the transgene by PCR and its expression by RT-qPCR (**Supplementary Figure 3**). We selected the overexpression lines OE-21, OE-1, and OE-4 as lines with high, medium, and low expression levels of the transgene for subsequent experiments (**Supplementary Figure 3**). As *Lb1G04794* was highly expressed during the development of salt glands in *L. bicolor*, we first characterized trichome and root hair development.

**Figure 3:**
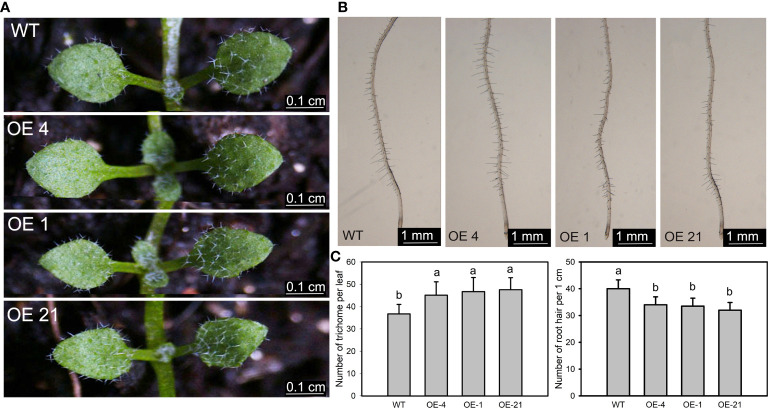
Trichome and root hair development in *35S*:*Lb1G04794* Arabidopsis lines. **(A)** Trichomes on the first two rosette leaves of the non-transgenic wild-type (WT, Col-0) and *35S*:*Lb1G04794* (OE 4, OE 1, and OE 21). Photographs show seedlings grown on half-strength MS medium for 7 days. Scale bar, 0.1 cm. **(B)** Root hairs in seedlings grown for 5 days on half-strength MS medium. Scale bar, 1 mm. **(C)** Number of trichomes per leaf (leaf) and root hairs (right) in WT and *35S*:*Lb1G04794* lines. Trichome number was counted in 10 seedlings. Root hair number was counted in the same region of each root (1 cm from root tip upward) for 10 seedlings per line. Data are means ± SD of 10 seedlings; different lowercase letters indicate significant differences at *P* = 0.05 according to Duncan’s multiple range test.

We counted the number of trichomes of the first fully extended true leaf of the Arabidopsis transgenic lines, which indicated that the overexpression lines have significantly more trichomes than the WT (**Figures 3A, C**). We identified *Lb1G04794* by screening for genes with high expression during salt gland development, underscoring the correlation between salt gland and trichome development. Similarly, we counted the number of root hairs over a 1-cm region from the root tip and observed that overexpression of *Lb1G04794* results in fewer root hairs, together with a shorter root, relative to the WT (**Figures 3B, C**).

### 3.5 Effect of NaCl treatment on the germination of Lb1G04794 overexpression Arabidopsis lines

**Figure 4:**
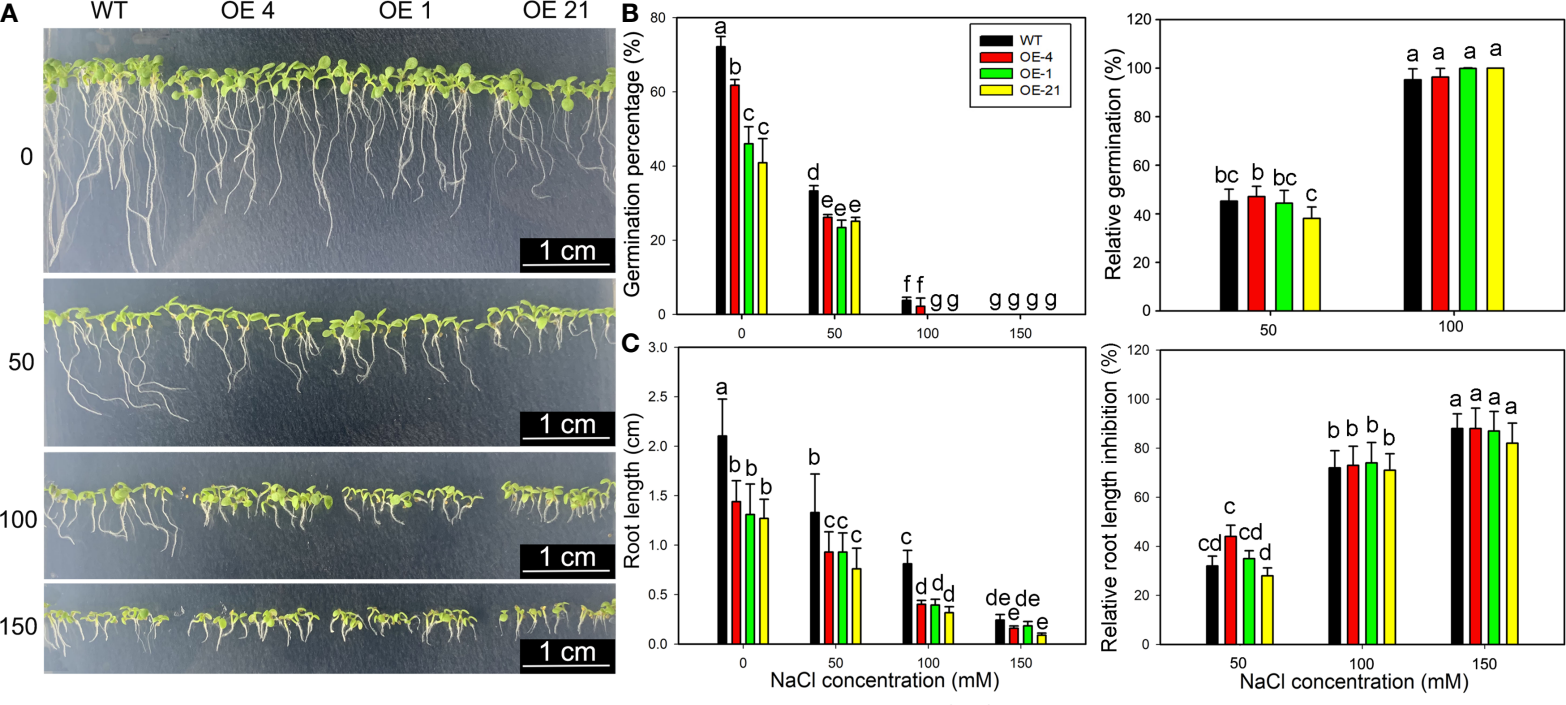
Growth characteristics of *35S:Lb1G04794* transgenic Arabidopsis lines following germination on different NaCl levels. **(A)** Phenotypes of WT and *35S:Lb1G04794* (OE 4, OE 1, and OE 21) Arabidopsis lines grown on half-strength MS medium (0) or different NaCl concentrations (50, 100, and 150 mM) for 5 days. **(B)** Germination percentages and germination inhibition percentages after 24 h at different concentrations of NaCl. The relative inhibition percentages of germination was calculated as (germination percentages under control – germination percentages under NaCl treatment)/germination percentages under control × 100%. **(C)** Root length and relative inhibition rate of root length elongation after 3 days at different concentrations of NaCl. The relative inhibition rate of root length elongation was calculated as (root length under control – root length under NaCl treatment)/root length under control × 100%. Fifty seeds per line were sown for each treatment, and three biological replicates were performed. The data for percentage germination are means ± SD. Root length of 5-day-old seedlings was measured in ImageJ. Data for root length are means ± SD of 10 seedlings per line; different lowercase letters indicate significant differences at *P* = 0.05 according to Duncan’s multiple range test.

Considering that *Lb1G04794* expression was induced by NaCl treatment (**Figure 1C**) and that fewer root hairs developed in *Lb1G04794* overexpression lines (**Figure 3B**), we explored salt tolerance–related indices at the germination stage in the transgenic lines. To this end, we sowed seeds from Arabidopsis and three transgenic lines onto medium containing different NaCl concentrations to determine the effect of NaCl on seed germination (**Figure 4A**). Notably, the germination of all *35S:Lb1G04794* overexpression lines was significantly lower under all conditions than in the WT of 24, 48, and 72 h after sowing, even when sown on control medium lacking added NaCl.

Although the germination percentage of the transgenic lines was lower than that of the WT (**Figure 4B**), the relative inhibition of root elongation (**Figure 4C**) was less affected by NaCl treatment than in the WT. These results indicated that germination and growth are inhibited upon overexpression of *Lb1G04794*, but the transgenic lines showed a greater salt tolerance than the WT under equivalent NaCl conditions.

The germination percentage of the transgenic lines was clearly inhibited under control (0 NaCl) conditions, which prompted us to measure the contents of endogenous phytohormones, with a focus on the three plant hormones IAA and ABA. We established that IAA contents in the overexpression lines are significantly lower than in the non-transgenic WT (**Supplementary Figure 4**), a result that was in agreement with the shorter roots of these lines, while the contents for ABA were similar between all genotypes. The lower IAA levels explained the shorter roots seen in the overexpression lines, indicating that *Lb1G04794* may negatively regulated IAA biosynthesis. These changes in plant hormone levels in the overexpression lines may reflect internal physiological changes of the overexpressing lines.

### 3.6 The effect of Lb1G04794 overexpression on salt tolerance at the seedling stage

**Figure 5:**
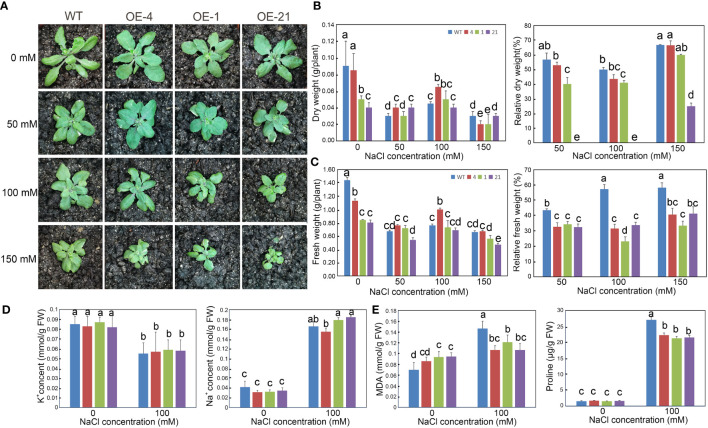
Growth status and determination of physiological indicators of Arabidopsis lines overexpressing *Lb1G04794* exposed to high-salt conditions. **(A)** Three-week-old plants of WT and 35S:*Lb1G04794* (OE 4, OE 1, and OE 21) lines under control conditions (0) or 50, 100, and 150 mM NaCl treatment. **(B, C)** Fresh **(C)** and dry weight **(B)** per plant of different lines under control conditions (0) or 50, 100, and 150 mM NaCl treatments. Relative reduction in fresh/dry weight per plant under control conditions (0) or 50, 100, and 150 mM NaCl treatments were also shown. **(D)** Na^+^ and K^+^ contents per plant under control conditions (0) or 100 mM NaCl treatment. **(E)** Contents for MDA or proline in each line under control conditions (0) or 100 mM NaCl treatment. Data are the means ± SD of three replicates; different lowercase letters indicate significant differences at *P* = 0.05 according to Duncan’s multiple range test.

We also investigated the long-term effect of NaCl treatment on the growth of Arabidopsis transgenic lines after a 2-week NaCl treatment in soil (**Figure 5A**). Although the biomass of the transgenic lines was lower than that of the WT, their relative dry and fresh weights appeared less affected by increasing NaCl concentrations than the WT (**Figures 5B, C**).

We also measured several physiological indices (Na^+^, K^+^, proline, and MDA contents) to explore the relationship between *Lb1G04794* and salt stress. Under 100 mM NaCl treatment, the proline contents of *35S*:*Lb1G04794* lines were lower than in WT. However, MDA contents in the overexpression lines under control conditions were higher than in the WT, suggesting that *Lb1G04794* overexpression imposes a degree of cellular stress in Arabidopsis. Na^+^ and K^+^ contents showed no significant differences between genotypes when treated with 100 mM NaCl (**Figure 5D**). MDA contents in the *35S:Lb1G04794* lines increased to a lesser extent than in the WT when exposed to 100 mM NaCl (**Figure 5E**).

### 3.7 Expression of salt tolerance marker genes in transgenic Arabidopsis overexpressing Lb1G04794

**Figure 6:**
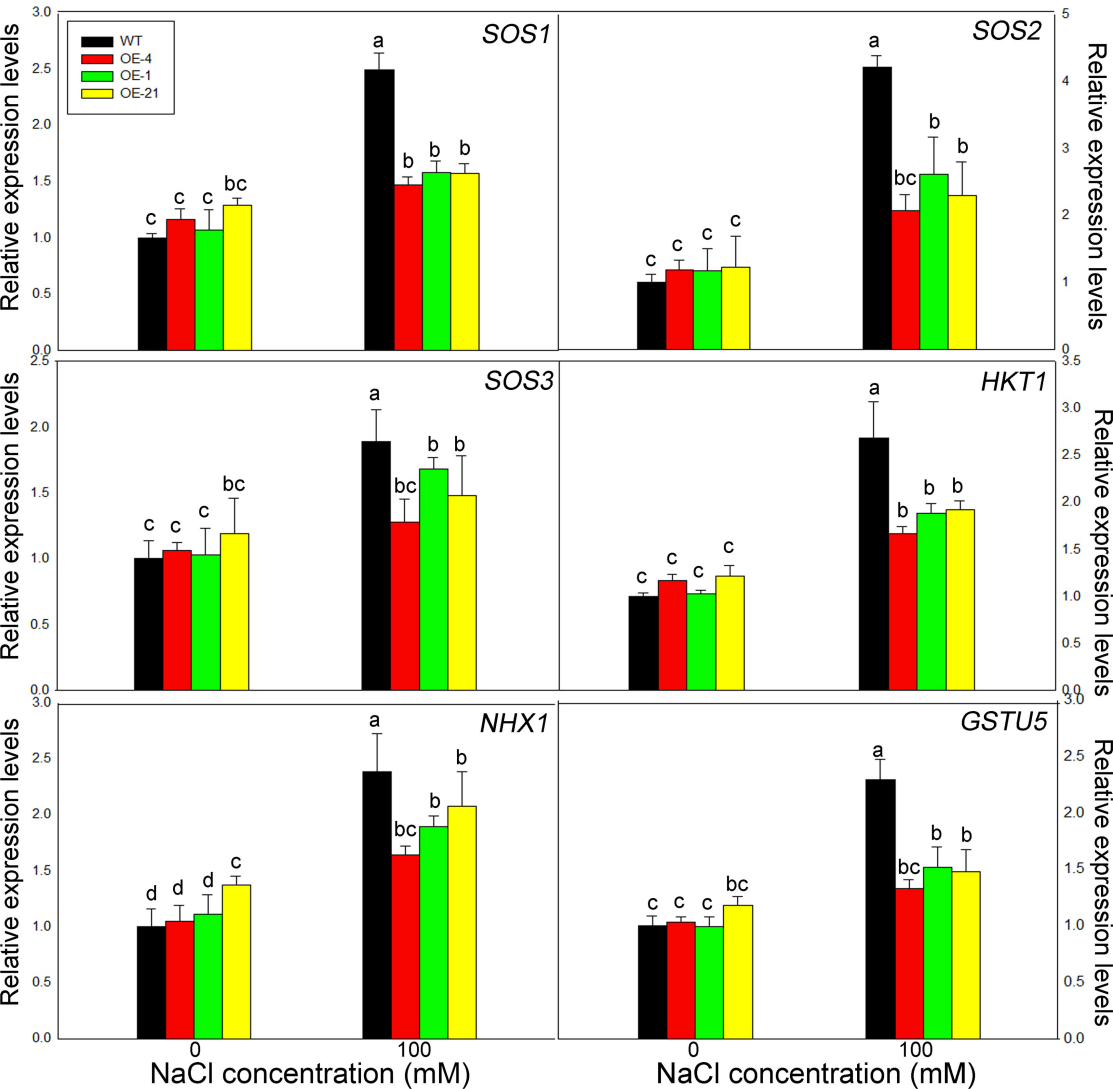
Expression levels of salt resistance–related marker genes in *Lb1G04794* transgenic Arabidopsis lines. Relative expression levels of *AtSOS1*, *AtSOS2*, *AtSOS3*, *AtHKT1*, *AtNHX1*, and *AtGSTU5*. WT and *35S:Lb1G04794* (OE 4, OE 1, and OE 21) Arabidopsis lines were grown for 7 days on half-strength MS medium (0) or with 100 mM NaCl. Data are means ± SD of three seedlings; different lowercase letters indicate significant differences at *P* = 0.05 according to Duncan’s multiple range test.

We determined the expression levels of the six salt tolerance marker genes *AtSOS1*, *AtSOS2*, *AtSOS3*, *AtHKT1*, *AtNHX1*, and *AtGSTU5* by RT-qPCR. Notably, all of these genes displayed the similar trends (**Figure 6**). Under control conditions, all six genes showed the same expression levels in the WT and the overexpression lines, with little significant difference between genotypes. Upon treatment with 100 mM NaCl, however, the transcript levels of all salt stress–related marker genes increased significantly in all genotypes, although the expression levels in the overexpression lines were significantly lower than those in the WT.

## 4 Discussion

Recretohalophytes *L. bicolor* is a typical salt-secreting halophyte with epidermal salt glands and can grow under high-salt conditions (Yuan et al., 2018), providing a very important repertoire of potential salt tolerance genes (Zhang et al., 2022). Here, *Lb1G04794* was an uncharacterized gene cloned from *L. bicolor*, which was highly expressed during salt gland development and participate in promoting salt gland differentiation by overexpression (**Figures 1**, **2**). The transgenic Arabidopsis lines have enhanced salt tolerance at the germination and seedling stage by reducing their salt sensitivity (**Figures 4**, **5**). Lb1G04794 localized in the nucleus with self-activation activity, which may act as a transcription factor to regulate the expression of related genes and further regulate plant adaptation to stress, especially salt stress.

As the most important and special structure of *L. bicolor* resistant to salt stress (Yuan et al., 2022), salt gland development were proposed by various genes. The overexpression of *Lb1G04794* in *L. bicolor* induced enhanced salt gland development (**Figure 2**), indicating that this gene positively regulate salt gland differentiation by promoting the initiation and development of salt glands to increase the number of salt glands in order to better adapt to high salt environment.

The salt resistance mechanisms of *Lb1G04794* were further verified in Arabidopsis by heterologous expression. At germination stage (**Figure 4**), although absolute indicators at the germination and seedling stages were lower than WT, a closer inspection of the results indicated that the transgenic lines exhibit the same phenotypes regardless of NaCl status, in contrast to the WT, whose germination percentage and root elongation were strongly and severely affected by high-salt treatment. By contrast, fewer root hairs in the overexpression lines limits the uptake of Na^+^ under high-salinity conditions (**Figure 3**). Further determination of plant hormone contents indicated that IAA contents are lower in the overexpression lines, which would help explain the shorter roots observed in these lines. The repression of germination and growth of the transgenic lines could make them more adaptable to salt stress. However, the comparison of germination percentages at the seedling stage was established on the premise of similar germination conditions without salt treatment (Mwando et al., 2020). *Lb1G04794* clearly showed an effect on germination percentage when overexpressed even under normal growth conditions, which we interpret as a buffer and adaptation to adverse growth conditions. So *Lb1G04794* can down-regulate the root development of Arabidopsis in the germination stage and delay plant growth in order to reduce the salt absorption and promote the salt resistance of transgenic lines in order to make the individual better adapt to the salt stress.

At seedling stage (**Figure 5**), the overexpression strains also showed resistance to salt stress. Through the measurement of various physiological indexes, it was found that the overexpressed lines accumulated less Na^+^, MDA and proline compared with WT under the same NaCl conditions. On the one hand, the less Na^+^ accumulation may be related to the less absorption due to fewer root hairs development (**Figure 3**) in overexpression lines. On the other hand, the physiological indicators were all consistent with the hypothesis that less Na^+^ was taken up by the overexpression lines, while osmoregulatory substances accumulated to cope with salt stress. Interesting, we validated this result with the determination of expression levels for salt tolerance marker genes (**Figure 6**), which again were consistent with diminished responses to salt stress in the overexpression lines, likely caused by reduced salt uptake.

Notably, the overexpression of *Lb1G04794* in Arabidopsis resulted in an increase in the number of trichomes (**Figure 3**), which is reminiscent of other reports in which the overexpression of a gene encoding a WD40-repeat protein from *L. bicolor* increased salt resistance by promoting trichome development in Arabidopsis (Yuan et al., 2019a). The experimental results also confirm the evolutionary relatedness of salt glands and trichomes, forming salt glands or trichomes as two different options for gene regulation (Yuan et al., 2022). This result illustrates the close relationship between salt gland development and trichome formation. We propose that salt glands and trichomes constitute two possible developmental trajectories that share several key genes.

*Lb1G04794*, a gene of unknown function in *L. bicolor*, showed a correlation between salt gland and trichome development in Arabidopsis, in addition to several developmental roles. Based on the current report of *Lb1G04794* participating in salt gland development and salt resistance, more application and the characterization of more salt gland-related genes will continue to be explored in order to offer new ways to combat high salinity.

## Data availability statement

The original contributions presented in the study are included in the article/**Supplementary Material**. Further inquiries can be directed to the corresponding authors.

## Author contributions

FY designed the research; XJ and BZ performed the research; XJ and BZ analyzed the data; XJ and FY wrote the paper; FY and BW revised the paper. All authors contributed to the article and approved the submitted version.

## Funding

This study was supported by the MOE Layout Foundation of Humanities and Social Sciences (21YJAZH108), and the National Natural Science Research Foundation of China (NSFC, project nos. 31770288 and 31600200).

## Conflict of interest

The authors declare that the research was conducted in the absence of any commercial or financial relationships that could be construed as a potential conflict of interest.

## Publisher’s note

All claims expressed in this article are solely those of the authors and do not necessarily represent those of their affiliated organizations, or those of the publisher, the editors and the reviewers. Any product that may be evaluated in this article, or claim that may be made by its manufacturer, is not guaranteed or endorsed by the publisher.

## Supplementary material

Supplementary Figure 1 The sequence and bioinformatics characteristics of *Lb1G04794*.

Supplementary Figure 2 Phylogenetic tree of *Lb1G04794* and related unknown proteins identified by NCBI-BLAST.

Supplementary Figure 3 Screening of transgenic Arabidopsis lines by PCR and RT-qPCR. **(A)** Genotyping results of Arabidopsis transgenic lines harboring the *35S*:*Lb1G04794* transgene. M, DNA marker with standard molecular weight of 2,000; lanes 1–15, different *35S*:*Lb1G04794* Arabidopsis lines; lane 16, negative control, with Col-0 DNA as template. **(B)** Relative expression levels of *Lb1G04794* in the Arabidopsis transgenic lines, as determined by RT-qPCR in *35S*:*Lb1G04794* lines; OE numbers represent the different *35S:Lb1G04794* overexpression lines. Data are means of three replicates ± SD; different lowercase letters indicate significant differences at *P* = 0.05 according to Duncan’s multiple range test.

Supplementary Figure 4 Determination of IAA and ABA contents. WT and *35S:Lb1G04794* (OE 4, OE 1, and OE 21) Arabidopsis seedlings were grown for 7 days on half-strength MS medium. Three biological replicates were performed. Data are means ± SD of three seedlings; different lowercase letters indicate significant differences at *P* = 0.05 according to Duncan’s multiple range test.

Supplementary Table 1 Sequences of the primers used in this study.

## References

[B1] Boyer J. S. (1982). Plant productivity and environment. Science 218(4571), 443–448. doi: 10.1126/science.218.4571.443. 17808529

[B2] Clough S. J. Bent A. F. (1998). Floral dip: a simplified method for agrobacterium -mediated transformation of arabidopsis thaliana. Plant Journal 16(6), 735–743. doi: 10.1046/j.1365-313x.1998.00343.x. 10069079

[B3] Cui Y. (2021). Cloning and salt resistance analysis of LpNAC17 gene from lilium pumilum (Northeast Forestry University).

[B4] Gao Y. Zhao B. Jiao X. Chen M. Wang B. Yuan F. (2021). Coupled development of salt glands, stomata, and pavement cells in limonium bicolor. Front. Plant Sci. 12, 745422. doi: 10.3389/fpls.2021.745422 34956255 PMC8695552

[B5] Guo J. Dong X. Li Y. Wang B. (2020a). NaCl Treatment markedly enhanced pollen viability and pollen preservation time of euhalophyte suaeda salsa *via* up regulation of pollen development-related genes. J. Plant Res. 133, 57–71. doi: 10.1007/s10265-019-01148-0 31654246

[B6] Guo J. Li Y. Han G. Song J. Wang B. S. (2017). NaCl Markedly improved the reproductive capacity of the euhalophyte suaeda salsa. Funct. Plant Biol. 44, 350–361. doi: 10.1071/fp17181. 32290958

[B7] Guo J. Lu C. Zhao F. Gao S. Wang B. (2020b). Improved reproductive growth of euhalophyte suaeda salsa under salinity is correlated with altered phytohormone biosynthesis and signal transduction. Funct. Plant Biol. 47, 170–183. doi: 10.1071/FP19215. 31941563

[B8] Guo S. Xu Y. Liu H. Mao Z. Zhang C. Ma Y.. (2013). The interaction between OsMADS57 and OsTB1 modulates rice tillering *via* DWARF14. Nat. Commun. 4, 1566. doi: 10.1038/ncomms2542. 23463009 PMC3615354

[B9] Han G. Yuan F. Guo J. Zhang Y. Sui N. Wang B. (2019). AtSIZ1 improves salt tolerance by maintaining ionic homeostasis and osmotic balance in arabidopsis. Plant Sci. 285, 55–67. doi: 10.1016/j.plantsci.2019.05.002 31203894

[B10] Hu B. Jin J. Guo A.-Y. Zhang H. Luo J. Gao G. (2015). GSDS 2.0: an upgraded gene feature visualization server. Bioinf. (Oxford England) 31, 1296–1297. doi: 10.1093/bioinformatics/btu817 PMC4393523 25504850

[B11] Jing X. (2021). Cloning and functional study of unknown genes Lb2G14763, Lb3G18904 and Lb7G32827 in limonium bicolor (Shandong Normal University).

[B12] Leng B. Geng F. Dong X. Yuan F. Wang B. (2019a). Sodium is the critical factor leading to the positive halotropism of the halophyte limonium bicolor. Plant Biosyst. - Int. J. Dealing all Aspects Plant Biol. 153, 544–551. doi: 10.1080/11263504.2018.1508085

[B13] Leng B. Wang X. Yuan F. Zhang H. Lu C. Chen M.. (2021). Heterologous expression of the limonium bicolor MYB transcription factor LbTRY in arabidopsis thaliana increases salt sensitivity by modifying root hair development and osmotic homeostasis. Plant Sci. 302, 110704. doi: 10.1016/j.plantsci.2020.110704. 33288017

[B14] Leng B. Y. Yuan F. Dong X. X. Wang J. Wang B. S. (2018). Distribution pattern and salt excretion rate of salt glands in two recretohalophyte species of limonium (Plumbaginaceae). South Afr. J. Of Bot. 115, 74–80. doi: 10.1016/j.sajb.2018.01.002.

[B15] Leng B. Zhao P. Dong X. Yuan F. Wang B. (2019b). Study on the physiological mechanism of early flowering and low Male fertility of limonium bicolor mutant vrl15. J. Plant Growth Regul. 38, 1206–1214. doi: 10.1007/s00344-019-09925-w

[B16] Lescot M. Déhais P G. Thijs G. Marchal K. Moreau Y. Van De Peer Y. Rouzé P.. (2002) PlantCARE, a database of plant cis-acting regulatory elements and a portal to tools for in silico analysis of promoter sequences. Nucleic Acids Res 30 (1362-4962), 7. doi: 10.1093/nar/30.1.325 PMC99092 11752327

[B17] Li J. Yuan F. Liu Y. Zhang M. Liu Y. Zhao Y.. (2020). Exogenous melatonin enhances salt secretion from salt glands by upregulating the expression of ion transporter and vesicle transport genes in limonium bicolor. BMC Plant Biol. 20, 493. doi: 10.1186/s12870-020-02703-x. 33109099 PMC7590734

[B18] Lu C. Feng Z. Yuan F. Han G. Guo J. Chen M.. (2020). The SNARE protein LbSYP61 participates in salt secretion in limonium bicolor. Environ. Exp. Bot. 176. doi: 10.1016/j.envexpbot.2020.104076.

[B19] Lu X. Liu R. Liu H. Wang T. Li Z. Zhang L.. (2022). Experimental evidence from suaeda glauca explains why the species is not naturally distributed in non-saline soils. Sci. Total Environ. 817, 153028. doi: 10.1016/j.scitotenv.2022.153028 35026244

[B20] Ma Y. Yang Y. Liu R. Li Q. Song J. (2020). Adaptation of euhalophyte suaeda salsa to nitrogen starvation under salinity. Plant Physiol. Biochem. 146, 287–293. doi: 10.1016/j.plaphy.2019.11.025 31783204

[B21] Munns R. Tester M. (2008). Mechanisms of salinity tolerance. Annu Rev Plant Biol 59, 651–681. doi: 10.1146/annurev.arplant.59.032607.092911 18444910

[B22] Murashige T. Skoog F. (1962). A revised medium for rapid growth and bio assays with tobacco tissue cultures. Physiol Plant 15(3), 473–497. doi: 10.1111/j.1399-3054.1962.tb08052.x.

[B23] Mwando E. Han Y. Angessa T. T. Zhou G. Hill C. B. Zhang X. Q.. (2020). Genome-wide association study of salinity tolerance during germination in barley (Hordeum vulgare l.). Front. Plant Sci. 11, 118. doi: 10.3389/fpls.2020.00118. 32153619 PMC7047234

[B24] Rasool S. Hameed A. Azooz M. M. Muneeb U R. Siddiqi T. O. Ahmad P. (2013). “Salt stress: Causes, types and responses of plants,” in Ecophysiology and responses of plants under salt stress, 1–24.

[B25] Song Y. Li J. Sui Y. Han G. Zhang Y. Guo S.. (2020). The sweet sorghum SbWRKY50 is negatively involved in salt response by regulating ion homeostasis. Plant Mol. Biol. 102, 603–614. doi: 10.1007/s11103-020-00966-4 32052233

[B26] Song J. Wang B. (2015). Using euhalophytes to understand salt tolerance and to develop saline agriculture: Suaeda salsa as a promising model. Ann. Bot. 115 (3). doi: 10.1093/aob/mcu194. PMC4332605 25288631

[B27] Sui N. Tian S. Wang W. Wang M. Fan H. (2017). Overexpression of glycerol-3-Phosphate acyltransferase from suaeda salsa improves salt tolerance in arabidopsis 8 (14). doi: 10.3389/fpls.2017.01337 PMC5539759 28824673

[B28] Sun W. Cao Z. Li Y. Zhao Y. Zhang H. (2007). A simple and effective method for protein subcellular localization using agrobacterium-mediated transformation of onion epidermal cells. Biologia 62, 529–532. doi: 10.2478/s11756-007-0104-6

[B29] Su M. Wang S. Liu W. Yang M. Zhang Z. Wang N.. (2022). Interaction between MdWRKY55 and MdNAC17-l enhances salt tolerance in apple by activating MdNHX1 expression. Plant science 320, 111282. doi: 10.1016/j.plantsci.2022.111282 35643619

[B30] Wang X. Wang B. Yuan F. (2022). Lb1G04202, an uncharacterized protein from recretohalophyte limonium bicolor, is important in salt tolerance. Int. J. Mol. Sci. 23 (10), 16. doi: 10.3390/ijms23105401 PMC9140551 35628211

[B31] Wang X. Zhou Y. Xu Y. Wang B. Yuan F. (2021). A novel gene LbHLH from the halophyte limonium bicolor enhances salt tolerance *via* reducing root hair development and enhancing osmotic resistance. BMC Plant Biol. 21, 284. doi: 10.1186/s12870-021-03094-3 34157974 PMC8218485

[B32] Wei L. (2006). Functional identification of an unknown gene in the EST database of saltmustard (Shandong Normal University).

[B33] Xu Y. Jiao X. Wang X. Zhang H. Wang B. Yuan F. (2021). Importin-β from the recretohalophyte limonium bicolor enhances salt tolerance in arabidopsis thaliana by reducing root hair development and abscisic acid sensitivity. Front. Plant Sci. 11, 2100. doi: 10.3389/fpls.2020.582459 PMC7838111 33519843

[B34] Yuan F. Chen M. Leng B. Y. Wang B. S. (2013). An efficient autofluorescence method for screening limonium bicolor mutants for abnormal salt gland density and salt secretion. South Afr. J. Bot. 88, 110–117. doi: 10.1016/j.sajb.2013.06.007.

[B35] Yuan F. Guo J. Shabala S. Wang B. (2018). Reproductive physiology of halophytes: Current standing. Front. Plant Sci. 9, 1954. doi: 10.3389/fpls.2018.01954. 30687356 PMC6334627

[B36] Yuan F. Leng B. Wang B. (2016a). Progress in studying salt secretion from the salt glands in recretohalophytes: How do plants secrete salt? Front. Plant Sci. 7, 977. doi: 10.3389/fpls.2016.00977. 27446195 PMC4927796

[B37] Yuan F. Leng B. Zhang H. Wang X. Han G. Wang B. (2019a). A WD40-repeat protein from the recretohalophyte limonium bicolor enhances trichome formation and salt tolerance in arabidopsis. Front. Plant Sci. 10, 1456. doi: 10.3389/fpls.2019.01456 31781150 PMC6861380

[B38] Yuan F. Liang X. Li Y. Yin S. Wang B. (2019b). Methyl jasmonate improves tolerance to high salt stress in the recretohalophyte limonium bicolor. Funct. Plant Biol. 46, 82–92. doi: 10.1071/FP18120 30939260

[B39] Yuan F. Lyu M. J. Leng B. Y. Zheng G. Y. Feng Z. T. Li P. H.. (2015). Comparative transcriptome analysis of developmental stages of the limonium bicolor leaf generates insights into salt gland differentiation. Plant Cell Environ. 38, 1637–1657. doi: 10.1111/pce.12514. 25651944

[B40] Yuan F. Lyu M.-J. A. Leng B.-Y. Zhu X.-G. Wang B.-S. (2016b). The transcriptome of NaCl-treated limonium bicolor leaves reveals the genes controlling salt secretion of salt gland. Plant Mol. Biol. 91 (3), 241–256. doi: 10.1007/s11103-016-0460-0. 26936070

[B41] Yuan F. Wang X. Zhao B. Xu X. Shi M. Leng B.. (2022). The genome of the recretohalophyte limonium bicolor provides insights into salt gland development and salinity adaptation during terrestrial evolution. Mol. Plant 15, 1024–1044. doi: 10.1016/j.molp.2022.04.011 35514085

[B42] Zhang M. Chen Z. Yuan F. Wang B. Chen M. (2022). Integrative transcriptome and proteome analyses provide deep insights into the molecular mechanism of salt tolerance in limonium bicolor. Plant Mol. Biol. 108, 127–143. doi: 10.1007/s11103-021-01230-z 34950990

[B43] Zhao X. (2020). Preliminary study on the involvement of ChAOX2 gene in salt resistance (Northeast Forestry University).

[B44] Zheng H. Yang Z. Wang W. Guo S. Li Z. Liu K.. (2020). Transcriptome analysis of maize inbred lines differing in drought tolerance provides novel insights into the molecular mechanisms of drought responses in roots. Plant Physiol. Biochem. 149, 11–26. doi: 10.1016/j.plaphy.2020.01.027. 32035249

[B45] Zhu M. (2014). A preliminary study of an unknown functional gene involved in osmotic stress response in arabidopsis thaliana (Nanjing university).

