# Supplementary figures and images for: An uncharacterized gene Lb1G04794 from Limonium bicolor promotes salt tolerance and trichome development in Arabidopsis
Source: Front Plant Sci. 2022 Dec 8;13:1079534. doi: 10.3389/fpls.2022.1079534 (PMC9773991; doi:10.3389/fpls.2022.1079534)

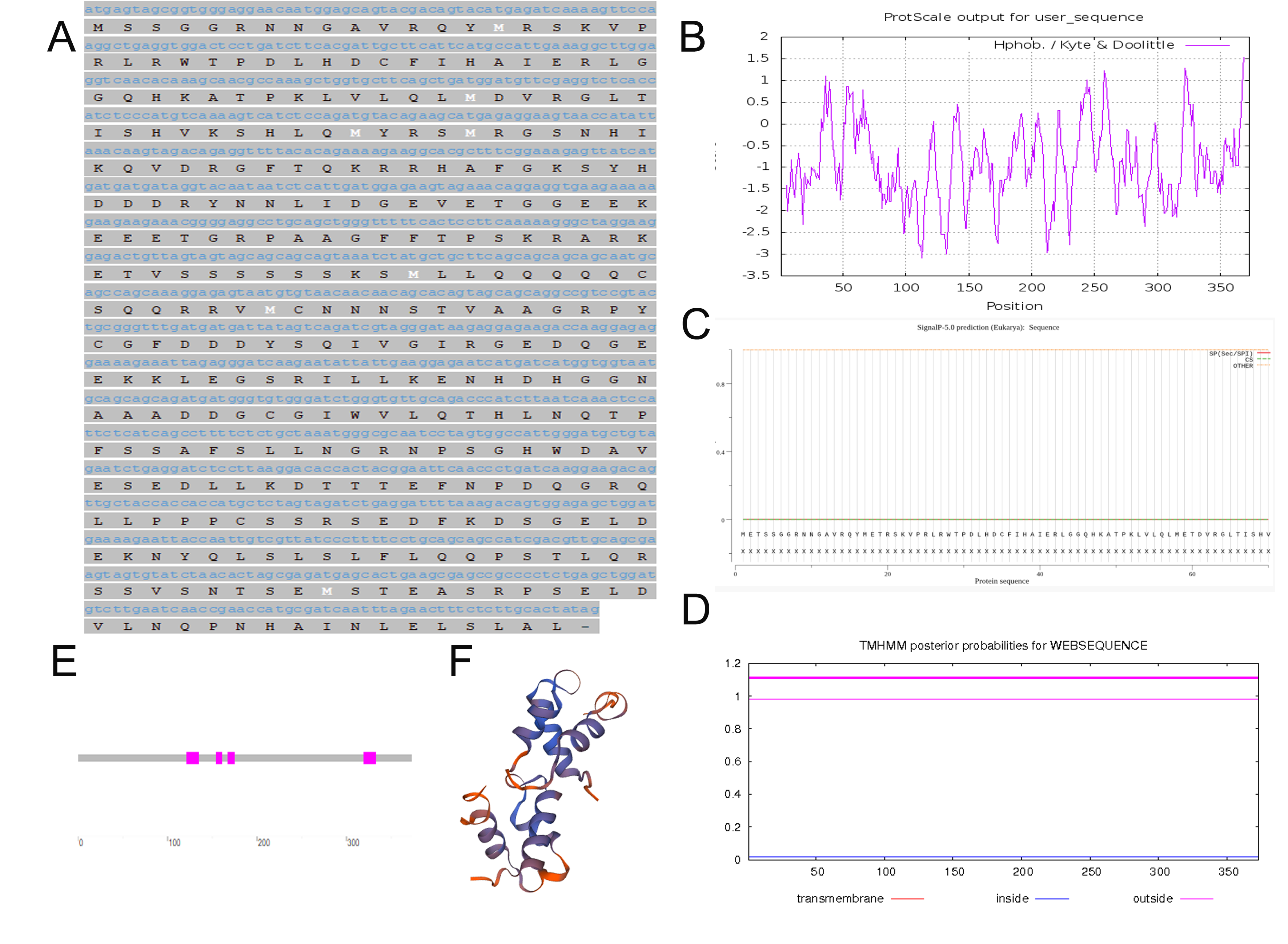

Supplement: Supplementary Figure 1 — The sequence and bioinformatics characteristics of Lb1G04794. [file Image_1.tif]

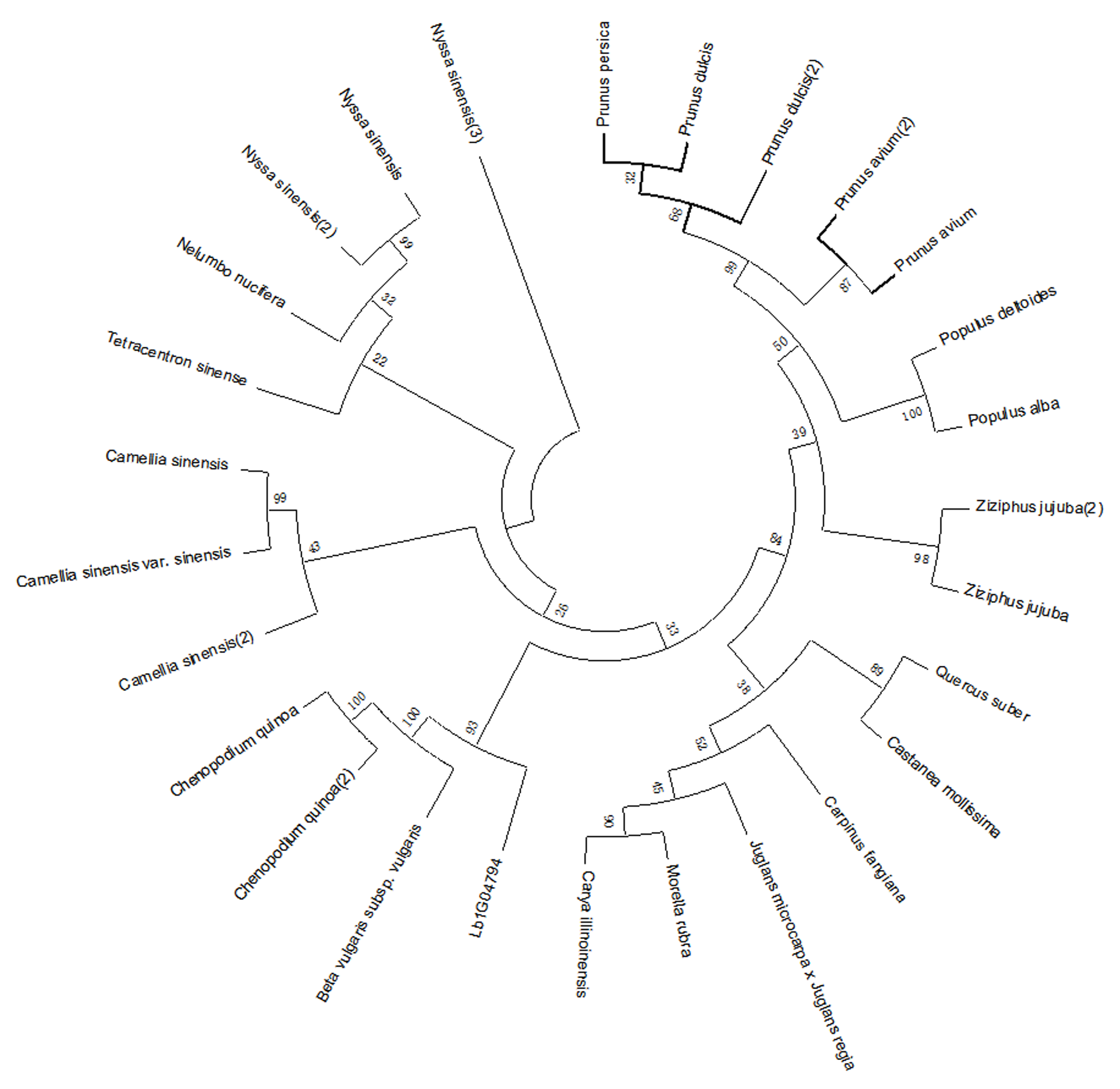

Supplement: Supplementary Figure 2 — Phylogenetic tree of Lb1G04794 and related unknown proteins identified by NCBI-BLAST. [file Image_2.tif]

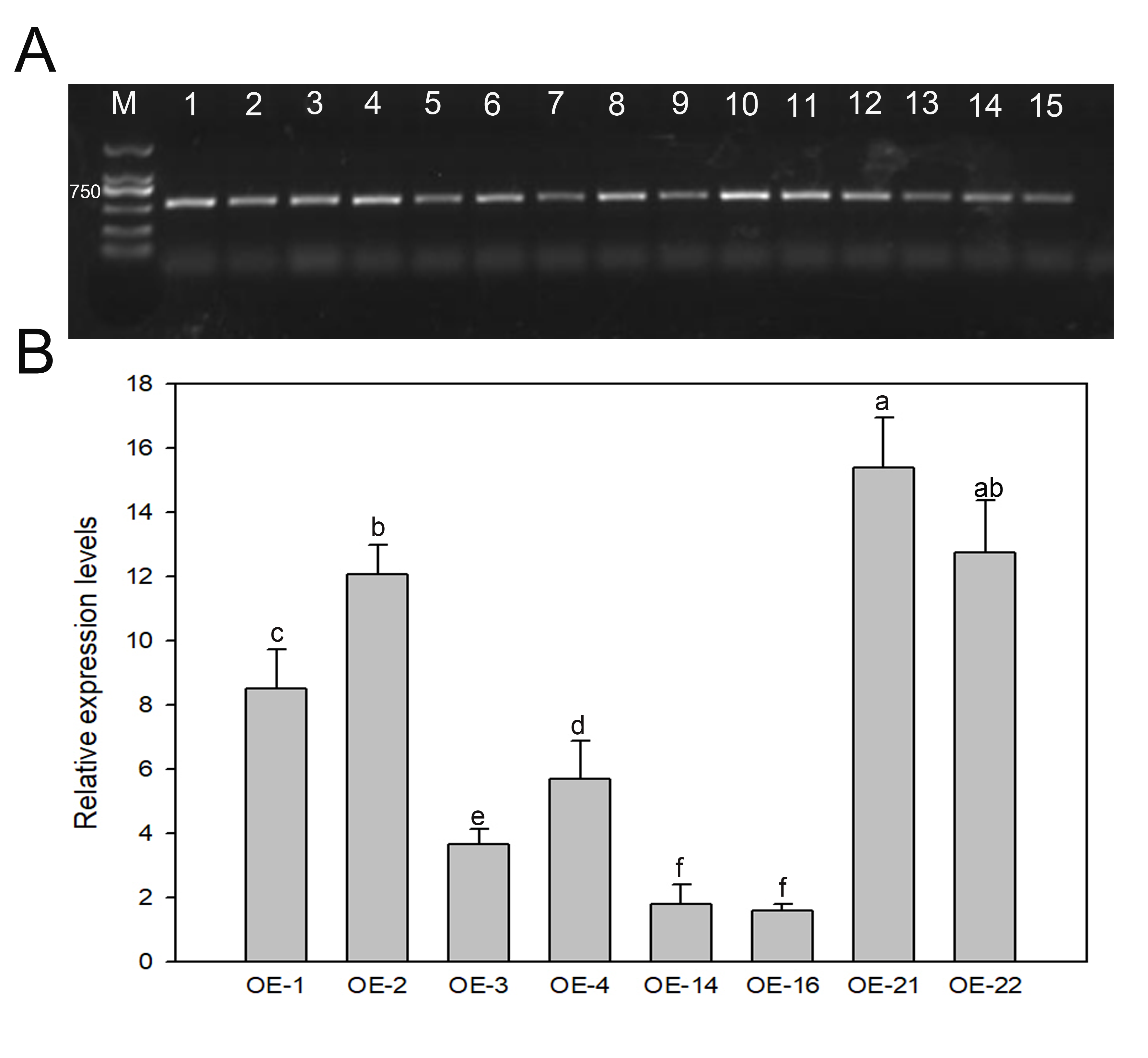

Supplement: Supplementary Figure 3 — Screening of transgenic Arabidopsis lines by PCR and RT-qPCR. (A) Genotyping results of Arabidopsis transgenic lines harboring the 35S:Lb1G04794 transgene. M, DNA marker with standard molecular weight of 2,000; lanes 1–15, different 35S:Lb1G04794 Arabidopsis lines; lane 16, negative control, with Col-0 DNA as template. (B) Relative expression levels of Lb1G04794 in the Arabidopsis transgenic lines, as determined by RT-qPCR in 35S:Lb1G04794 lines; OE numbers represent the different 35S:Lb1G04794 overexpression lines. Data are means of three replicates ± SD; different lowercase letters indicate significant differences at P = 0.05 according to Duncan’s multiple range test. [file Image_3.tif]

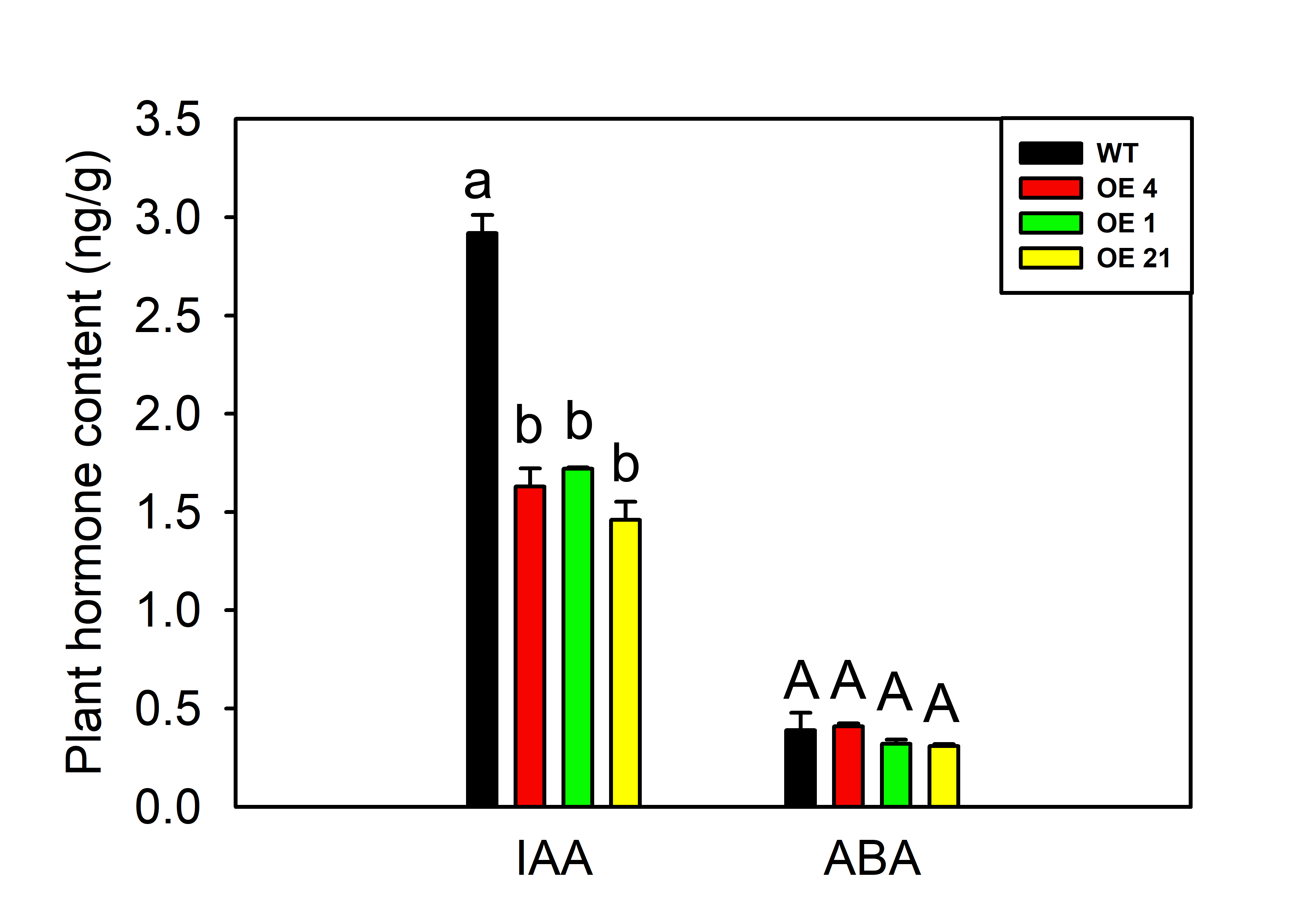

Supplement: Supplementary Figure 4 — Determination of IAA and ABA contents. WT and 35S:Lb1G04794 (OE 4, OE 1, and OE 21) Arabidopsis seedlings were grown for 7 days on half-strength MS medium. Three biological replicates were performed. Data are means ± SD of three seedlings; different lowercase letters indicate significant differences at P = 0.05 according to Duncan’s multiple range test. [file Image_4.tif]
