# Supplementary material for: An uncharacterized gene Lb1G04794 from Limonium bicolor promotes salt tolerance and trichome development in Arabidopsis
Source: Front Plant Sci. 2022 Dec 8;13:1079534. doi: 10.3389/fpls.2022.1079534 (PMC9773991; doi:10.3389/fpls.2022.1079534)
Supplement: Supplementary Table 1 — Sequences of the primers used in this study. [file Table_1.docx]

**Supplemental Table 1** The primers used in this study

| **Name** | **Oligonucleotide sequence** | **Role** |
| --- | --- | --- |
| Lb1G04794-S | 5-AGGAGAGTAATGTGTAACA-3 | Full length amplification |
| Lb1G04794-A | 5-CTACGATCTGACTATAATCATC-3 |  |
| Lb1G04794-P-S | 5-TTATAGATTGTGGTGTTATTAGTGGTG-3 | Full promoter length amplification |
| Lb1G04794-P-A | 5-TTTCTCTCTCGTGTATGGCTCAAC-3 |  |
| Lb1G04794 3301-P-S | 5-acgggggactcttgaccatggTTATAGATTGTGGTGTTATTAGTGGTG-3 | Construction of p35S::Lb1G04794-promoter-GUS vectors |
| Lb1G04794 3301-P-A | 5-ttaccctcagatctaccatggTTTCTCTCTCGTGTATGGCTCAAC-3 |  |
| Lb1G04794 3301-S | 5-acgggggactcttgaccatggATGAGTAGCGGTGGGAGGAAC-3 | Construction of p35S::Lb1G04794-GUS vectors digested with NcoI |
| Lb1G04794 3301-A | 5-ttaccctcagatctaccatggCTATAGTGCAAGAGAAAGTTCTAAATTGAT-3 |  |
| Lb1G04794 1300-S | 5-cggggatcctctagagtcgacATGAGTAGCGGTGGGAGGAAC-3 | Construction of p35S::Lb1G04794-GFP vectors digested with SalⅠ |
| Lb1G04794 1300-S | 5-gcccttgctcaccatgtcgacCTATAGTGCAAGAGAAAGTTCTAAATTGAT-3 |  |
| Lb1G04794 BD-S | 5-tcagaggaggacctgcatatgATGAGTAGCGGTGGGAGGAAC-3 | Construction of pGBKT7-*Lb1G04794* vectors digested with NdeⅠ |
| Lb1G04794 BD-A | 5-ttcggcctccatggccatatgTAGTGCAAGAGAAAGTTCTAAATTGATC-3 |  |
| PCAMBIA-S | 5-CTAGAAATTTACTAACACATGC-3 | Identification of overexpressed lines |
| Lb1G04794-A | 5-CTACTTCTCCATCAATGAGATTAT-3 |  |
| Lb1G04794-RT-S | 5-TAACAACAACAGCACAGTA-3 | Real-time PCR in different developmental stages, condition  and Arabidopsis overexpression  lines |
| Lb1G04794-RT-A | 5-TGGTCTTCTCCTCTTATCC-3 |  |
| ACTIN2 sense | 5-GGTAACATTGTGCTCAGTGGTGG-3 |  |
| ACTIN2 anti | 5-AACGACCTTAATCTTCATGCTGC-3 |  |
| LbTUBULIN-S | 5-GGTTGAGTGAGCAGTTCAC-3 |  |
| LbTUBULIN-A | 5-GATAACCAGCCACACCTTAGC-3 |  |
| AtSOS1-RT-S | 5-ATTTTGATGCAGTCAGTGGATG-3 | RT-qPCR verification of osmotic  response related marker genes in  Col-35S:: Lb1G04202 |
| AtSOS1-RT-A | 5-GCAAGCAGATTCTAGTCTTTCG-3 |  |
| AtSOS2-RT-S | 5-GCGAACTCAATGGGTTTTAAGT-3 |  |
| AtSOS2-RT-A | 5-CTTACGTCTACCATGAAAAGCG-3 |  |
| AtSOS3-RT-S | 5-CCGGTCCATGAAAAAGTCAAAT-3 |  |
| AtSOS3-RT-A | 5-CTCTTTCAATTCTTCTCGCTCG-3 |  |
| AtHKT1-RT-S | 5-CCTCTACGTCTCCTATTTCACC-3 |  |
| AtHKT1-RT-A | 5-ACTAAGAACCACCGAGTACAAG-3 |  |
| AtNHX1-RT-S | 5-GTTGCCCTTATGATGCTTATGG-3 |  |
| AtNHX1-RT-A | 5-TTCTTGAGCTCTCCGTTACATT-3 |  |
| AtGSTU5-RT-S | 5-GTACGTGGAAGAGATACTGGAG-3 |  |
| AtGSTU5-RT-A | 5-CAAGAACAGGGACTTTCTTGTG-3 |  |
